# Post‐hatching growth of the limbs in an altricial bird species

**DOI:** 10.1002/vms3.357

**Published:** 2020-09-16

**Authors:** Jianjian Yan, Zihui Zhang

**Affiliations:** ^1^ College of Life Sciences Capital Normal University Beijing China

**Keywords:** altricial, limb bone, ontogenetic allometry, pigeon

## Abstract

The fore‐ and hindlimbs of birds are specialized to perform different functions. The growth patterns of limb bones and their relationship with the ontogeny of locomotion are critical to our understanding of variation in morphological, physiological and life‐history traits within and among species. Unfortunately, the ontogenetic development of limb bones has not been well explored, especially in altricial birds. In this study, we sampled the entire measurements of the pigeon (*Columba livia*) of individual skeletons, to investigate the ontogenetic allometry of limb bones by reduced major axis regression. The ulna and humerus were found to be positively allometric in relation to body mass, with the ulna growing more rapidly than the humerus. Together with previous data, this suggests that strong positive allometric growth in forelimb bones could be a common trend among diverse Carinatae groups. Hindlimb was dominated by positive allometry, but was variable in the growth of the tarsometatarsus which included three allometric patterns. A greater dorsoventral diameter in the midsection of the humerus and ulna confers superior bending resistance and is ideal for flapping/gliding flight. Shape variation in the midsection of different hindlimb components reflects different mechanical loading, and the markedly inverse trend between the tibiotarsus and tarsometatarsus before 28 days of age also suggests loading change before fledging. Before fledging, the growth of the leg bones was prior to that of the wing bones. This kind of asynchronous development of the fore‐ and hindlimbs was associated with the establishment and improvement of different functions, and with shifts in the importance of different functions over time.

## Graphical abstract

Post‐natal growth of limb bones was less explored, especially for altricial birds. We use detailed measurements, including width and depth of the proximal and distal ends, rather than the traditional focus on the length and midshaft width to investigate the ontogenetic allometry of the pigeon (*Columba livia*). The results of this study provide valuable information in poultry management, and are necessary for kinetics and kinematics studies.

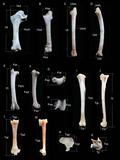

## 1 INTRODUCTION

Variation in prenatal development leads avian hatchlings to differ markedly in the degree of maturation of behaviour, physiology and anatomy, which determine the extent of hatchling dependence on parental care. Based on the wide range of variation in the above‐mentioned factors, neonates of different bird species can be categorized along the complicated altricial‐precocial spectrum (Starck & Ricklefs, 1998). Chicks of altricial species hatch in an almost embryo‐like state, are characterized in blind, naked, less developed locomotion organs and a dependence on parental care immediately after hatching. At the other extreme, the young of precocial species hatch covered in down with eyes open; their locomotive organs are well developed, and they are highly self‐sufficient feeders. The different morphologies of precocial and altricial neonates at hatching are thought to be associated with different patterns of embryonic development (Blom & Lilja, 2005). Many comparative studies on prenatal development in birds have revealed developmental heterochrony in the head, brain, eyes, limbs and digestive organs among precocial and altricial species. Regarding the locomotive organs, for example, in the altricial fieldfare (*Turdus pilaris*), the forelimb buds were significantly larger than the hindlimb buds at Hamburger–Hamilton (HH) stages 18–25 of embryonic development, but in the precocial quail (*Coturnix japonica*), there were no size differences between the fore‐ and hindlimb buds at corresponding stages (Blom & Lilja, 2005). In the barn owl (*Tyto alba*), legs were slightly larger than the wings during the early embryonic development, but approximately equal in length later (Kӧppl, Futterer, Nieder, Sistermann, & Wagner, 2005). In the ostrich (*Struthio camelus*) and emu (*Dromaius novaehollandiae*), the hindlimbs were formed with a slight advance in developmental timing and were considerably larger than forelimbs due to a higher growth rate since then (Nagai et al., 2011). In the pigeon (*Columba livia*), the development of the limbs was asynchronous, with the forelimb buds appearing earlier (H.H. stage 16) and reaching a larger relative size than the hindlimb in more time before hatching (Olea, Hernando, & Lombardo, 2016; Olea & Sandoval, 2012). As in chicken (Hamburger & Hamilton, 1951), the forelimb buds appeared at HH stage 16 in ducks (*Anas platyrhynchos*) and geese (*Anser cygnoides*), a little earlier than their leg buds, but remained relatively small throughout subsequent embryonic development until hatching (Li et al., 2019).

Limbs are not subject to functional demands before hatching, but undergo different developmental patterns as mentioned above. After hatching, they are crucial for foraging, behavioural thermoregulation and escape from predation (Hayward, Henson, Banks, & Lyn, 2008). Additionally, altricial species grow more rapidly than precocial species (Ricklefs, 1979). These complicated factors are expected to influence the post‐natal ontogenetic development of limb bones, which is reported to present different allometric patterns among species; nevertheless, this kind of works, especially detailed studies, are relatively rare (Picasso, 2012), and only investigated in ostrich, black noddy, California gull, greater rhea, mallard and Japanese quail (Bennett, 2008; Carrier & Leon, 1990; Dial & Carrier, 2012; Picasso, 2012; Ren, Wang, & Zhang, 2016; Smith, Jespers, & Wilson, 2010), all of which are precocial/semiprecocial taxa. The aim of the present study, therefore, is to reveal the limb growth pattern of an altricial bird species and to compare it with that of precocial birds. Domestic pigeons (*C. livia*) are widely used as experimental models in the study of aerodynamics, kinematics, physiology and navigation of flight (Taylor, Portugal, & Biro, 2017). It is usually known as altricial, and sometimes assigned to semi‐altricial because their hatchlings are covered in downy plumage. Unlike precocial hatchlings, however, pigeon chicks are unable to move until 5 days of age (Olea et al., 2016), and after which point they use their legs to compete with siblings and for other in‐nest activities. These activities, although low intensity, also will pose functional requirement to their legs. Pigeon chicks grow rapidly and begin to stretch and exercise their wings at the age of about 3 weeks, 1 week before they leave the nest. We thus predict that the functional maturity of the wings is likely to lag behind that of the legs, and that rapid growth and functional performance are not compatible. Because wings are only responsible for flight, which was established later in function than the legs, we expected the scaling slopes for forelimbs to be similar and positively allometric; leg dimensions, on the other hand, are predicted to show early maturity and slower growth than those of the wing, and to exhibit mixed growth patterns to satisfying complicated interaction of mechanical demands and other factors.

## 2 MATERIALS AND METHODS

**FIGURE 1:**
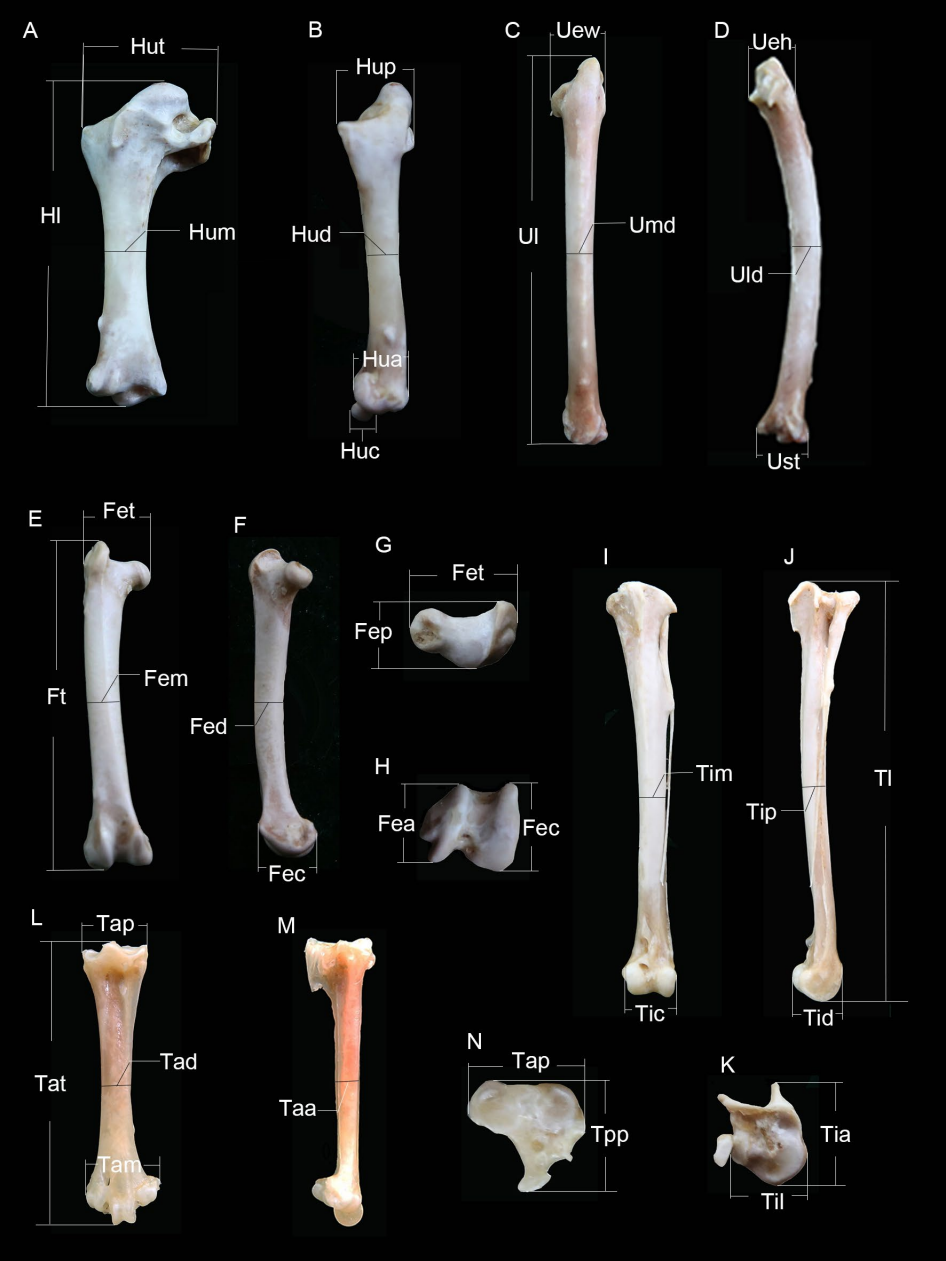
Measurements of the limb bones. Not to scale. (a, b) Left humerus in caudal (a) and dorsal (b) views; Hl, humerus length; Hum, dorsoventral diameter of midshaft; Hud, anteroposterior diameter of midshaft; Hut, dorsoventral width of proximal end; Hup, anteroposterior width of proximal end; Hua, anteroposterior width of condylus dorsalis; Huc, anteroposterior width of condylus ventralis. (c, d) Left ulna in caudal (c) and dorsal (d) views; Ul, ulna length; Umd, dorsoventral diameter of midshaft; Uld, anteroposterior diameter midshaft; Uew, dorsoventral width of proximal end; Ueh, anteroposterior width of proximal end; Ust anteroposterior width of distal end. (e–h) Right femur in anterior (e), medial (f), proximal (g) and distal (h) views; Ft, femoral length; Fem, mediolateral diameter of midshaft; Fed, anteroposterior diameter of midshaft; Fet, mediolateral width of proximal end; Fep, anteroposterior width of proximal end; Fea, anteroposterior width of condylus lateralis; Fec: anteroposterior width of condylus medialis. (i–k) Left tibiotarsus in anterior (i), lateral (j) and proximal (k) views; Tl, tibiotarsus length; Tim, mediolateral diameter of midshaft; Tip, anteroposterior diameter of midshaft; Tia, anteroposterior width of proximal end; Til, mediolateral width of proximal end; Tic, mediolateral width of distal end; Tid: anteroposterior width of distal end. (l, m) Right tarsometatarsus in anterior (l), lateral (m) and proximal (n) views; Tat, tarsometatarsus length; Tad, mediolateral diameter of midshaft; Taa, anteroposterior diameter of midshaft; Tap, mediolateral width of proximal end; Tpp, anteroposterior width of proximal end; Tam, mediolateral width of distal end

A breed of domestic pigeon, the silver king pigeon, was used for the present study. They were incubated naturally, fed by both parents until the age of 30 days and subsequently reared in nursery and flying loft, following the 'technical regulation of feeding and management for meat pigeon' issued by the China Animal Agriculture Association. A total of 100 pigeons were collected at the ages of 4, 7, 14, 21, 28, 56, 112, 168, 252 and 336 days post‐hatching from a commercial pigeon farm in Beijing. The samples represented 10 stages of growth, and each stage contained 10 individuals. Body mass was measured with a digital balance (0.01 g precision) before decapitation. This research protocol was approved by the Animal Care and Ethics Committee of my university. Skeletal specimens were prepared after whole wings and legs were isolated from the carcasses by dissection. The muscle and surrounding soft tissue were then carefully removed from the bones; cartilage on epiphyseal areas was kept in its original site and shape. Out of the need for future mechanical research, all limb bones were wrapped in gauze, soaked in saline solution and frozen at −20°C. After thawing, a total of 33 linear measurements (Figure 1) were made with a 150‐mm digital caliper (0.01 mm precision); for immature individuals, unossified epiphyses were included in the measurements of bone length as well as width of proximal and distal ends. Legs of birds are responsible for body support; thus, the size and shape of articular surfaces can reflect limb weight‐bearing properties, as well as being strongly correlated with joint mobility, mode of locomotion and activity level (Ruff, 1988). The width and depth of the proximal and distal ends of the hindlimb bones were measured and treated as indicators of articular dimension. Besides articular morphology, a limb bone's resistance to mechanical loading may be reflected in the diaphyseal cross‐sectional geometry, such as the ratio of diameters along anatomical axes (Carlson & Judex, 2007). In the present study, midshaft diameters along the dorsoventral (DV) and anteroposterior (AP) axes of the forelimb bones, as well as the mediolateral (ML) and AP of the hindlimb were measured. The average of each measurement and average body mass for each stage were calculated. To investigate the relationship between body mass and skeletal measurements, log10 transformed means for each age group were used to perform reduced major axis regression using SMATR (Standardized Major Axis Tests and Routines) (v.2.0) software (Falster, Warton, & Wrigth, 2006). Growth was considered to be isometric when the scaling exponent (slope) was not significantly different from 0.333 at the 5% significance level (bone dimension versus. body mass). 5% confidence intervals above or below isometry and *p* < .05 were considered to be positively or negatively allometric, respectively.

## 3 RESULTS

The mean body masses of the animals in the study ranged from 59 to 560 g (Table 1). Raw data for wing and leg bones are shown in Tables 2 and 3, respectively. The exponents, correlation coefficients, *y*‐intercepts and confidence intervals, as well as the results of isometry test for all measurements are summarized in Table 4. Compared with the hindlimb bones, the humerus and ulna were relatively shorter, <30% of adult length at the age of 4 days (Table 2). All measurements of the humerus and ulna increase relatively fast than corresponding variables of the hindlimb, displaying strong positive allometry relative to body mass (Table 4). Differences in slope indicated that overall, the relative growth rate of the ulna, such as the total length (Ul), and the robustness of the bone (Umd and Uld) were more rapid than that of the humerus. Among three leg bones, the tarsometatarsus was relatively the longest at the age of 4 days, accounting for about 42% of adult length, followed by the femur and the tibiotarsus; the length of the tibiotarsus increased relatively faster among hindlimb bones, evidenced by the fact that this bone had the highest slope (0.52). Apart from distal tibiotarsal width (Tic), all measurements of the femur and the tibiotarsus presented positive allometry. The growth of the tarsometatarsus was complicated, exhibiting three different growth patterns: the length (Tat), the AP width of the midshaft (Taa) and proximal end (Tpp) for positive allometry, the ML width of the midshaft (Tad) and proximal end (Tap) for isometry and the distal width (Tam) for negative allometry (Table 4).

**TABLE 1:** Body mass of the pigeon

Age (days)	Body mass (mean ± *SD*, g)
4	59.12 ± 9.31
7	95.41 ± 12.71
14	360.37 ± 40.98
21	479.20 ± 44.97
28	389.31 ± 17.39
56	410.38 ± 30.81
112	521.00 ± 62.08
168	560.00 ± 59.25
252	512.38 ± 47.03
336	496.65 ± 57.08

**TABLE 2:** Measurements of the humerus and ulna (mean ± *SD*) (mm)

Age (days)	Humerus							Ulna					
	Hl	Hum	Hud	Hut	Hup	Hua	Huc	Ul	Umd	Uld	Ueh	Uew	Ust
4	15.29 ± 1.19	1.76 ± 0.28	1.63 ± 0.20	5.76 ± 0.54	3.36 ± 0.28	2.82 ± 0.50	2.45 ± 0.46	16.60 ± 1.28	1.37 ± 0.28	1.18 ± 0.16	2.52 ± 0.31	3.04 ± 0.28	2.39 ± 0.41
7	21.03 ± 1.21	2.53 ± 0.23	2.07 ± 0.23	8.71 ± 0.79	4.53 ± 0.19	3.71 ± 0.36	2.97 ± 0.54	22.46 ± 1.73	1.82 ± 0.21	1.61 ± 0.17	3.23 ± 0.37	4.22 ± 0.46	3.22 ± 0.45
14	40.23 ± 1.91	4.76 ± 0.20	3.54 ± 0.20	14.32 ± 1.38	7.02 ± 0.87	5.27 ± 0.71	3.92 ± 0.65	45.86 ± 2.78	3.58 ± 0.25	3.31 ± 0.23	4.87 ± 0.52	6.36 ± 0.49	5.13 ± 0.52
21	47.56 ± 1.27	5.42 ± 0.49	4.35 ± 0.44	16.67 ± 5.92	8.67 ± 0.60	6.52 ± 0.31	4.55 ± 0.26	55.51 ± 1.89	4.22 ± 0.29	3.91 ± 0.27	5.80 ± 0.28	8.10 ± 0.39	6.43 ± 0.46
28	48.73 ± 2.13	5.71 ± 0.16	4.31 ± 0.20	19.98 ± 0.85	9.59 ± 0.69	7.40 ± 0.48	5.14 ± 0.35	57.61 ± 2.58	4.28 ± 0.26	3.91 ± 0.38	6.13 ± 0.56	8.83 ± 0.54	6.73 ± 0.32
56	49.32 ± 1.83	5.57 ± 0.17	4.24 ± 0.18	20.18 ± 0.68	9.53 ± 0.57	7.63 ± 0.36	5.66 ± 0.51	57.66 ± 2.61	4.30 ± 0.23	3.80 ± 0.17	6.15 ± 0.27	9.11 ± 0.42	6.84 ± 0.58
112	51.51 ± 2.01	6.20 ± 0.37	4.83 ± 0.26	21.85 ± 0.92	10.54 ± 0.42	8.58 ± 0.47	6.49 ± 0.37	59.95 ± 2.39	4.66 ± 0.26	4.34 ± 0.22	7.06 ± 0.36	10.03 ± 0.69	7.70 ± 0.49
168	51.88 ± 1.89	6.24 ± 0.37	4.95 ± 0.38	21.50 ± 1.61	11.00 ± 0.72	8.74 ± 0.42	7.03 ± 0.60	60.67 ± 1.85	4.90 ± 0.60	4.43 ± 0.40	7.40 ± 0.51	10.11 ± 0.44	7.52 ± 0.29
252	51.93 ± 1.38	6.22 ± 0.19	4.88 ± 0.25	20.45 ± 2.60	11.99 ± 0.68	8.50 ± 0.35	5.99 ± 0.57	60.65 ± 1.49	4.84 ± 0.45	4.40 ± 0.33	7.28 ± 0.34	9.92 ± 0.27	7.51 ± 0.45
336	50.93 ± 2.38	6.31 ± 0.37	4.87 ± 0.33	20.71 ± 1.36	11.15 ± 0.82	8.50 ± 0.51	6.36 ± 1.00	59.53 ± 2.63	4.72 ± 0.49	4.65 ± 0.39	7.11 ± 0.44	9.55 ± 0.55	7.14 ± 0.45

**TABLE 3:** Measurements of leg bones (mean ± *SD*) (mm)

Age	Femur						
(days)	Ft	Fem	Fed	Fet	Fep	Fea	Fec
4	18.51 ± 2.03	1.59 ± 0.15	1.73 ± 0.14	5.46 ± 0.78	3.44 ± 0.51	3.90 ± 0.46	3.93 ± 0.45
7	25.32 ± 1.43	2.17 ± 0.15	2.08 ± 0.16	6.67 ± 0.41	4.28 ± 0.19	4.97 ± 0.33	4.95 ± 0.53
14	41.32 ± 1.39	3.57 ± 0.19	3.11 ± 0.13	8.16 ± 0.52	4.54 ± 1.62	5.71 ± 0.43	5.68 ± 0.46
21	45.38 ± 1.51	3.89 ± 0.15	3.48 ± 0.13	9.59 ± 0.32	5.60 ± 0.27	6.38 ± 0.26	6.27 ± 0.23
28	44.96 ± 2.06	3.87 ± 0.23	3.51 ± 0.20	9.60 ± 0.47	5.69 ± 0.38	6.74 ± 0.42	6.64 ± 0.48
56	45.59 ± 1.69	3.89 ± 0.17	3.53 ± 0.21	9.55 ± 0.50	5.49 ± 0.35	6.85 ± 0.49	6.71 ± 0.36
112	47.03 ± 1.82	4.31 ± 0.23	4.04 ± 0.23	10.74 ± 0.48	6.41 ± 0.27	7.74 ± 0.42	7.59 ± 0.61
168	47.29 ± 1.88	4.33 ± 0.33	4.18 ± 0.32	10.80 ± 0.64	6.37 ± 0.31	7.78 ± 0.42	7.70 ± 0.38
252	46.82 ± 0.99	4.21 ± 0.16	4.01 ± 0.16	10.49 ± 0.51	6.32 ± 0.43	7.56 ± 0.30	7.36 ± 0.48
336	45.80 ± 2.06	4.19 ± 0.24	4.06 ± 0.28	10.41 ± 0.60	6.29 ± 0.62	7.69 ± 0.51	7.61 ± 0.53

Tibiotarsus							
	Tl	Tim	Tip	Tia	Til	Tic	Tid
4	20.91 ± 1.66	1.79 ± 0.13	1.60 ± 0.12	3.61 ± 0.34	2.95 ± 0.44	3.43 ± 0.30	2.68 ± 0.33
7	27.71 ± 1.35	2.34 ± 0.13	1.90 ± 0.10	5.35 ± 0.40	4.92 ± 0.48	5.06 ± 0.24	3.80 ± 0.31
14	52.18 ± 2.36	3.88 ± 0.24	2.94 ± 0.15	8.92 ± 0.65	7.18 ± 0.67	7.79 ± 0.30	6.04 ± 0.38
21	61.68 ± 2.05	4.11 ± 0.29	3.23 ± 0.24	9.27 ± 0.55	7.30 ± 0.35	7.83 ± 0.26	6.75 ± 0.26
28	62.66 ± 2.64	4.27 ± 0.22	3.24 ± 0.17	10.11 ± 0.36	7.52 ± 0.26	7.67 ± 0.37	6.85 ± 0.28
56	62.50 ± 2.05	4.33 ± 0.17	3.26 ± 0.18	10.21 ± 0.40	7.67 ± 0.39	7.55 ± 0.21	6.91 ± 0.18
112	64.65 ± 2.37	4.52 ± 0.28	3.63 ± 0.23	10.54 ± 0.41	8.65 ± 0.38	7.76 ± 0.36	7.40 ± 0.34
168	65.28 ± 1.73	4.86 ± 0.42	3.73 ± 0.34	10.79 ± 0.71	8.56 ± 0.64	8.22 ± 0.63	7.61 ± 0.37
252	65.11 ± 1.70	4.61 ± 0.32	3.72 ± 0.22	10.05 ± 0.72	8.28 ± 0.40	8.23 ± 0.33	7.37 ± 0.35
336	64.17 ± 2.89	4.57 ± 0.31	3.85 ± 0.31	10.23 ± 0.81	8.27 ± 0.48	8.10 ± 0.51	7.44 ± 0.42

Tarsometatarsus						
	Tat	Tad	Taa	Tap	Tpp	Tam
4	15.07 ± 1.21	1.84 ± 0.20	1.28 ± 0.14	3.88 ± 0.43	2.26 ± 0.30	4.94 ± 0.90
7	18.66 ± 1.09	2.42 ± 0.18	1.73 ± 0.21	5.52 ± 0.26	3.88 ± 0.36	6.26 ± 0.24
14	29.59 ± 1.25	3.76 ± 0.20	2.82 ± 0.09	8.34 ± 0.27	6.86 ± 0.53	7.44 ± 0.39
21	34.49 ± 1.28	3.99 ± 0.14	3.10 ± 0.25	8.75 ± 0.49	7.99 ± 0.41	7.96 ± 0.52
28	34.30 ± 1.52	3.50 ± 0.18	3.00 ± 0.22	8.17 ± 0.61	7.63 ± 0.40	8.26 ± 0.32
56	34.48 ± 1.33	3.51 ± 0.24	3.18 ± 0.19	8.02 ± 0.43	7.62 ± 0.27	7.98 ± 0.67
112	35.77 ± 1.19	3.97 ± 0.26	3.52 ± 0.25	8.72 ± 0.60	8.09 ± 0.49	8.62 ± 0.28
168	35.76 ± 1.79	3.93 ± 0.22	3.32 ± 0.20	9.08 ± 0.39	8.45 ± 0.42	8.90 ± 0.28
252	35.35 ± 0.76	3.86 ± 0.20	3.11 ± 0.19	8.41 ± 0.41	8.01 ± 0.24	8.65 ± 0.48
336	35.56 ± 1.67	3.86 ± 0.36	3.36 ± 0.38	8.84 ± 0.66	8.07 ± 0.40	8.93 ± 0.53

**TABLE 4:** Results of allometric analyses and test of isometry

Bone	Variables	Allometric analysis						Test of isometry	
		*R* ^2^	*p*	Slope	LowCI	UppCI	Interc	*F*	*p*
Humerus	Hl	.986	.000	0.55+	0.50	0.61	0.21	164.311	.000
	Hum	.984	.000	0.56+	0.51	0.62	−0.73	160.665	.000
	Hud	.945	.000	0.50+	0.45	0.55	−0.68	88.090	.000
	Hut	.945	.000	0.58+	0.48	0.69	−0.24	49.927	.000
	Hup	.936	.000	0.54+	0.44	0.66	−0.44	32.134	.000
	Hua	.915	.000	0.50+	0.40	0.64	−0.45	17.687	.003
	Huc	.975	.000	0.65+	0.58	0.74	−0.95	176.255	.000
Ulna	Ul	.984	.000	0.60+	0.54	0.66	0.17	199.178	.000
	Umd	.990	.000	0.57+	0.53	0.62	−0.87	257.270	.000
	Uld	.989	.000	0.60+	0.55	0.66	−0.99	296.744	.000
	Ueh	.951	.000	0.47+	0.39	0.56	−0.44	21.113	.002
	Uew	.972	.000	0.66+	0.58	0.76	−0.87	158.745	.000
	Ust	.965	.000	0.51+	0.44	0.60	−0.53	47.326	.000
Femur	Ft	.983	.000	0.41+	0.37	0.46	0.56	24.020	.001
	Fem	.990	.000	0.43+	0.40	0.47	−0.54	58.664	.000
	Fed	.973	.000	0.38+	0.34	0.44	−0.45	6.862	.031
	Fet	.980	.000	0.49+	0.44	0.55	−0.30	64.565	.000
	Fep	.967	.000	0.42+	0.36	0.49	−0.35	14.984	.005
	Fea	.947	.000	0.48+	0.40	0.58	−0.42	22.426	.001
	Fec	.948	.000	0.47+	0.39	0.56	−0.39	19.072	.002
Tibiotarsus	Tl	.986	.000	0.52+	0.47	0.58	0.40	128.155	.000
	Tim	.986	.000	0.42+	0.39	0.47	−0.49	37.282	.000
	Tip	.976	.000	0.38+	0.34	0.43	−0.48	7.240	.027
	Tia	.967	.000	0.46+	0.40	0.54	−0.23	28.160	.001
	Til	.940	.000	0.43+	0.35	0.52	−0.24	9.321	.016
	Tic	.953	.000	0.36=	0.31	0.43	−0.07	1.603	**.241**
	Tid	.982	.000	0.44+	0.40	0.50	−0.34	40.566	.000
Tarsometatarsus	Tat	.986	.000	0.40+	0.36	0.44	0.48	19.439	.002
	Tad	.973	.000	0.33=	0.29	0.38	−0.30	0.000	**.990**
	Taa	.981	.000	0.42+	0.38	0.47	−0.62	26.462	.001
	Tap	.963	.000	0.35=	0.30	0.41	0.01	0.615	**.455**
	Tpp	.971	.000	0.55+	0.48	0.63	−0.57	78.981	.000
	Tam	.939	.000	0.24−	0.20	0.29	0.29	14.064	.006

Note

Value marked with + indicates positive allometry, − negative allometry, = isometry. Isometry testing showed that the slopes of Tic, Tad and Tap were not significantly different from 0.33. Bold values indicate tests for isometry were not significantly from 0.333.

Abbreviations: CI, confidence interval; Interc, intercept.

Regarding changes in shaft diameter, the diameters of the humerus and the ulna were initially smaller than those of leg bones, but underwent more rapid growth and became greater upon sexual maturity (Table 2). With the exception of the ML diameter of the tarsometatarsus, the growth of all diameters was positively allometric. Furthermore, the diameters of the humerus and ulna exhibited higher slopes than corresponding variables of the hindlimb bones (Table 4). Direct measurements suggested that wing bones experienced rapid growth with age until 21 days, at which time the humerus increased by 2.1‐ and 1.7‐fold in the DV and AP directions, respectively; corresponding increases in the ulna were 2.1‐ and 2.3‐fold. Meanwhile, the cross‐sectional shapes of both humerus and ulna were elongated in the DV direction (Table 2), but with different growth patterns; the humerus became more elliptical, as evidenced by a change in the ratio of DV diameter/AP diameter from 1.08 to 1.25, but the change was reversed for the ulna (ratio change from 1.16 to 1.08). From the age of 3 weeks, the humerus and the ulna grew gradually and reached their highest value at 168 days (Table 2). The increase in diameters was very similar in the femur and the tibiotarsus, both were higher in the slope of the ML diameter than that of the AP diameter. On the contrary, the tarsometatarsus scaled with the highest positive allometry in its AP diameter among the hindlimb bones. Growth in diameters of the femur, tibiotarsus and tarsometatarsus was relatively lower than that of wing bones, increased less than 1.5‐fold until 21 days post‐hatching. All leg bones, especially the tibiotarsus and tarsometatarsus, were larger in ML diameter than in AP diameter (Table 3). The change in the ratio of ML diameter/AP diameter suggests that the midsection of the femur was more circular than those of the distal leg bones. The overall shape of the midshaft in the tibiotarsus and tarsometatarsus was mediolaterally elongated, but showed markedly inverse trend before 28 days by the fact that the ratio of ML diameter/AP diameter increased from 1.1 to 1.3 in the tibiotarsus, but decreased from 1.4 to 1.2 in the tarsometatarsus.

Compared with the increases in diameters along the AP and ML axes, the growth of articular surfaces at the distal end of the femur, both ends of the tibiotarsus and the proximal end of the tarsometatarsus was relatively more rapid. The slopes of the AP and ML width of the proximal femur were also higher than those of the diameters.

## 4 DISCUSSION

### 4.1 Fore‐ and hindlimb growth differences

Avian fore‐ and hindlimbs are diversified in function, with forelimbs usually being employed for flight. The forelimbs and hindlimbs of most birds arise and mature coincident with one another. Anseriformes, Charadriiformes, Gruiformes, Gaviiformes and Podicipediformes present asynchronous development in their wings and legs. Birds that develop asynchronously, such as mallard, California gull and black noddy, show strong positive allometry in forelimb bones (Bennett, 2008; Carrier & Leon, 1990; Dial & Carrier, 2012). In our previous work, Japanese quails, which develop synchronously, were also shown to undergo positively allometric growth in their humerus and ulna (Ren et al., 2016). Consistent with available data on the California gull, quail, mallard and black noddy, the growth of length of the humerus and ulna was characterized by strong positive allometry in pigeons in the present study. Furthermore, the ulna increased relatively fast than the humerus. Regardless of the developmental pattern (altricial or precocial) and differences in the onset of locomotion, strong positively allometric growth in forelimb bones could be a common trend among diverse Carinatae groups that have yet to be fully explored or recognized (Table 5). For pigeons, strong positive allometry and the relatively fast growth in the wing bones compared to the hindlimbs should be correlated with differences in the onset and intensity of activities involving fore‐ and hindlimbs. Growth patterns are variable in hindlimbs, for example, bone length presents isometry, positive and negative allometry among different species (Table 5). As suggested by Hayward et al. (2008), bone growth rates are determined by a complex suite of factors and interactions, many of which remain incompletely understood; furthermore, the lack of uniformity in the growth of leg bones might also be related to their complicated functions.

**TABLE 5:** Comparison of scaling pattern for bone length

Species	Femur	Tibiotarsus	Tarsometatarsus	Humerus	Ulna
Greater rhea *Rhea americana*	=	=	+		
Ostrich *Struthio camelus*	+	+	+		
California gull *Larus californicus*	=	+	+	+	+
Black noddy *Anous minutus*	−	+	−	+	+
Japanese quail *Coturnix coturnix japonica*	+	+	−	+	+
Mallard *Anas platyrhynchos*	=	−	=	+	+
Pigeon *Columba livia*	+	+	+	+	+

Note

Data from: Bennett, 2008; Carrier & Leon, 1990; Dial & Carrier, 2012; Picasso, 2012; Ren et al., 2016; Smith et al., 2010.

Abbreviations: −, negative allometry; +, positive allometry; =, isometry.

### 4.2 Increase in length and diameters

In the present study, the DV and AP diameters of the humerus and ulna increased at faster rates than body mass. Direct measurements also suggest constant and rapid growth in the length, and DV and AP diameters of the humerus and ulna until 21 days post‐hatching. Both the relative and absolute high growth rate of pigeons may be correlated with their altricial development, and may further coincided with the fact that pigeon nestlings begin to stretch and exercise their wings at the age of about 3 weeks, 1 week before they leave the nest (Liang, Yu, Wang, & Zhang, 2018); similarly, in other pigeons and doves, as nestlings grew older, they flap their wings more often and markedly before fledging (Thorsen, Innes, Nugent, & Prime, 2004). The ratio of DV diameter/AP diameter provides a measure of cross‐sectional shape, which may in turn reflect a bone's resistance to mechanical loading. In flying birds, the humerus is subjected to significant torsion and DV bending owing to lift forces acting on the wing during the downstroke; likewise, the ulna is subjected to bending (Biewener & Dial, 1995; Pennycuick, 1967). From the data recorded in the present study, the following morphological characteristics of forelimb in pigeon can be identified: (a) the midsection of the humerus and ulna was not circular, and was similar in their overall shape and growth pattern; and (b) both the humerus and ulna were elongated in the DV direction, which is a structural design that favours bending resistance. Birds are diverse in flight mode, which can be reflected in cross‐sectional shape of the wing bones (Marelli & Simons, 2014). As a flap‐gliding species (Tobalske & Dial, 1996), the characteristic of a more or less elliptical cross‐sectional shape of the pigeon is consistent with other flapping/soaring and flapping/gliding birds (Marelli & Simons, 2014). The pectoralis muscle is the most important flight muscle, and provides birds with lift and thrust for flight. During post‐natal development, this muscle presented marked increase in force‐production capacity (Liang et al., 2018), and as a result, will pose higher load to the humerus and the ulna.

Unlike the wing bones, increases in the length and diameters of the hindlimb bones were relatively slow, as evidenced by the slopes and direct measurements. This finding, consequently, may indicate that legs were more developed and have higher priority in function than wing bones, because pigeon chicks are altricial, hatching naked, blind and immobile; they acquire the ability to stand at about 5 days of age (Olea et al., 2016), whereupon they begin to use their legs to compete with siblings and for other in‐nest activities. These activities, although low intensity, also will pose functional requirements to their legs.

Hindlimbs of birds are not only responsible for body support and terrestrial movements, but also play an important role in flight during landing and take‐off. Leg bones experience axial, bending and twisting loads (Casinos & Cubo, 2001). By comparing the diameters along two axes of the leg bones, it was suggested that the midsection of the femur was more circular, in contrast to the mediolaterally elongated shape of the tibiotarsus and tarsometatarsus. This kind of shape difference may reflect different mechanical loadings among hindlimb components; for example, the femur is submitted to higher torsion moments than the other limb bones (Carrano & Biewener, 1999). A more circular femur is indicative of a constant level of function throughout development, and better resistance to torsional loads or bending in multiple directions rather than in one specific plane (Marelli & Simons, 2014). Consistent with the result of de Margerie, Sanchez, and Castanet (2005), who found that the tibiotarsi and tarsometatarsi are less circular in cross section, the two distal leg components of pigeon were elongated in the ML direction, suggesting high resistance to bending in the direction of the larger cross section (Simons, Hieronymus, & O’Connor, 2011). Although the overall shape was suggested similar in the tibiotarsus and the tarsometatarsus, they displayed different growth patterns; in particular, the negative allometry in ML diameter of the tarsometatarsus suggested shape change during development, from more elliptical to less elliptical in midsection, which further indicates loading change during ontogeny. Available data suggest that morphological specialization for different locomotor ecology is associated with functional shifts in gait that varies with leg morphology (Daley & Birn‐Jeffery, 2018). The characteristics and variation of the shape design in the tarsometatarsus should be consistent with the ontogeny of posture and gait, from an immobile nestling to a crouching young individual, and finally a relatively straight leg posture in adults; and also a reflection in the change of function. As we know, most of the initial acceleration during the first wingbeat is produced by the legs in pigeon (Berg Robertson & Biewener, 2012), and during take‐off, the tarsometatarsus displays a high amplitude of motions and is responsible for most of the propulsion. These facts may indicate that the tarsometatarsus suffer more complicated loading than other leg components during take‐off (Provini & Abourachid, 2018).

### 4.3 Joint surface increase in the hindlimb

Unlike shaft of limb bones, joints are primarily resisting compressive loads (Godfrey, Sutherland, Boy, & Gomberg, 1991); they have to adapt to the stresses and strains imposed by increasing body mass and movement throughout post‐natal ontogeny. Enlarged joint surfaces have biomechanical significance in weight‐bearing, as well as in the stability and mobility of the articulation (Swartz, 1989). While many studies have demonstrated varied scaling pattern in mammals, there is a lack of research on joint surfaces in birds. In the present study, linear measurements (e.g. ML and AP width), although not direct and accurate, were used to estimate joint surface areas. Our results show that the femoral condyle, both ends of the tibiotarsus, as well as the proximal depth of the tarsometatarsus scaled more strongly than their shaft diameters, reflecting more rapid increase in the articular surface of the knee and intertarsal joints, which helps the joints to withstand the relatively greater weight and higher loads imposed by the muscles that develop during ontogeny. As joint surface is irregular and three‐dimensioned, and proportional to body weight raised to the 2/3 power, new methods, such as surface scanning technology, will be necessary for future investigations.

## CONFLICT OF INTEREST

The authors declare no conflict of interest.

## AUTHOR CONTRIBUTION

**Jianjian Yan:** Data curation; Investigation; Methodology; Software; Writing‐original draft. **Zihui Zhang:** Funding acquisition; Investigation; Methodology; Project administration; Supervision; Visualization; Writing‐original draft; Writing‐review & editing.

## Data Availability

All relevant data are within the paper. The original data that support the findings of this study are available from the corresponding author upon reasonable request.
